# Red blood cell distribution width as a predictor of mortality in critically ill pediatric patients: a systematic review and meta-analysis

**DOI:** 10.3389/fped.2025.1646179

**Published:** 2025-09-15

**Authors:** Yinhong Yu, Xiaomei Hu, Yaping Shen

**Affiliations:** Department of Paediatrics, Shengzhou People's Hospital (the First Affiliated Hospital of Zhejiang University Shengzhou Branch), Zhejiang, China

**Keywords:** biomarker, death, critically ill, children, survival

## Abstract

**Objective:**

Red blood cell distribution width (RDW) has been found to predict outcomes in critically ill adult patients. However, its utility in pediatric patients remains unexplored. We reviewed published evidence and conducted a meta-analysis to assess whether RDW can be used to predict mortality in the pediatric intensive care unit (PICU).

**Methods:**

All observational studies assessing the association between RDW and PICU mortality available on the databases of PubMed, Embase, Scopus, and Web of Science up to 4th November 2024 were included. A detailed review of study outcomes was conducted with a meta-analysis.

**Results:**

Seven studies were included. 6,327 pediatric patients were included in these studies. On qualitative analysis, five of the seven studies found a statistically significant association between high RDW and PICU mortality. Four studies used RDW as a continuous variable, while three studies reported specific RDW cut-offs. Meta-analysis showed that an incremental increase in RDW was associated with a statistically significant increased risk of mortality (OR: 1.24 95% CI: 1.07, 1.44 I^2^ = 32%). Pooled analysis of studies using RDW as a categorical variable showed that higher values of RDW were associated with significantly higher risk of mortality (OR: 1.73 95% CI: 1.02, 2.92 I^2^ = 77%).

**Conclusions:**

RDW could be a potential predictor of mortality in the PICU. Results need to be interpreted with caution owing to the limited number of studies with variable study populations. Additional studies are needed to strengthen evidence.

**Systematic Review Registration:**

PROSPERO (CRD42024606208).

## Introduction

Pediatric intensive care units (PICUs) are an essential component of care of critically ill children with either respiratory distress, acute neurological deterioration, cardiovascular collapse, major infections, poisonings or any other life-threatening condition (1). About 13.4% of all pediatric patients require PICU admission and high-level of care (2), and these figures are only increasing (3). Mortality in PICU varies significantly and can range from just 2% to as high as 33% (1, 4). Age, severity of disease, and organ dysfunction are important factors that are predictive of mortality in children admitted to PICU (5). Furthermore, low- to middle-income countries usually have limited resources and fewer PICU services which in turn translates into higher mortality rates as compared to high-income countries (6). In China, the PICU mortality rate can be double or triple as compared to Western populations (4). Accurate prediction of mortality rates in the PICU can aid in therapeutic decision-making and allocation of resources. Such information can also be used to counsel caregivers and prioritize those with a higher risk of death (7). Currently, the most commonly used scores to predict mortality in PICU include the Pediatric Risk of Mortality Score (PRISM) III/IV, Pediatric Index of Mortality (PIM) 2/3 and Pediatric Logistic Organ Dysfunction (PELOD) 2 scores (4). While these scores have high accuracy, they cannot be easily calculated and do not provide a rapid prognostication of critically ill children (8). Furthermore, their use in a resource-limited setting may be difficult. Therefore, there is a need for simple, readily available markers that can provide a quick assessment of critically ill children.

Red blood cell distribution width (RDW) is a routinely measured hematological marker that is dependent on the circulating red blood cell volume. RDW is one of the most basic investigations conducted in all healthcare setups and can be measured by automated cell counters during blood counts. It basically measures the red blood cell size variation in the patient's sample and is generated from the distribution curve width and the mean cell size (9). Research shows that RDW can be a marker to predict outcomes in cardiac illnesses (like atrial fibrillation, heart failure, coronary heart disease), respiratory disorders like pulmonary embolism, sepsis, kidney and liver disease, stroke, and even in cancer patients (10–16). The advantage of the marker is its ease of availability and low cost, and hence can be a cost-effective way of rapid primary risk stratification of patients even with limited healthcare resources (17). Most of the studies examining the prognostic ability of RDW have been conducted in adult patients, especially with those in the intensive care unit (10–16). However, its use for pediatric patients has received limited attention. A separate investigation for pediatric patients is necessary because RDW varies between adults and children, with higher RDW values commonly reported in infants and newborns than in adults. RDW is larger in early life due to the dynamic nature of erythropoiesis, and gradually diminishes as a child grows older. RDW is much higher in infants, with reference intervals ranging between 14.2% and 17.8% in the first 30 days of life. RDW levels in children aged 3 months to 18 years tend to be greater than in adults, but lower than in newborns (18, 19). Given the gap in the literature, we will systematically review studies and conduct a meta-analysis to assess whether RDW can be used to predict mortality in the PICU.

## Material and methods

### Protocol registration

The review and meta-analysis are performed according to the PRISMA guidelines (20) which also includes pre-registration of the protocol on PROSPERO for transparency. The registration number on PROSPERO was: CRD42024606208.

### Identification of studies

We identified studies published in peer-reviewed journals using an electronic search of PubMed, Embase, Scopus, and Web of Science databases. Two authors were involved in the search, which identified articles published between the inception of these databases to 4th November 2024. All authors agreed and approved a broad search query which was formulated after discussions with a medical librarian who was an expert in database search. Combining both free-text and MeSH terms we used the following search strategy: ((((red cell distribution) OR (red blood cell distribution)) OR (RDW)) AND (((pediatric) OR (children)) OR (infants))) AND ((((mortality) OR (death)) OR (survival)) OR (length of hospital stay)). The terms “critical care” and “PICU” were not included in the search strategy in order to maximize sensitivity and avoid prematurely narrowing the pool of studies. Instead, eligibility for critical care populations was determined during the full-text screening phase. All databases were explored using the same strategy. The reviewers also found it pertinent to search Google Scholar and the reference lists of included studies to avoid any missed studies.

### Study selection

The inclusion criteria for the studies were devised based on PECOS. We included studies fulfilling these conditions: (1) The *population* was pediatric (<18 years) patients managed in PICU. (2) *The exposure* variable was high RDW. (3) *Comparison* group was low RDW. (4) *Outcomes* of interest included PICU mortality and length of PICU or hospital stay with at least one outcome being reported as adjusted summary estimates. (5) Observational *studies* published as full-length articles.

The reviewers excluded the following studies: (1) Studies conducted on neonates only. (2) Studies not separating data from adult patients. (3) Studies not exclusively on PICU patients. (4) Studies exclusively on post-surgical patients. (5) Studies with duplicate data.

The selection process from the literature search followed a clear pre-defined process. To avoid duplicity of articles, we first excluded all duplicates electronically. The remaining studies were then examined by the two authors one by one by reading titles and abstracts only. In this initial step, non-relevant studies were removed and all remaining studies were downloaded. In the last step, the full texts were read and cross-checked against the inclusion criteria. When both authors were satisfied, the study was included in the review. Otherwise, any disagreements were resolved after discussion with the third author.

### Risk of bias and data management

The quality of the included studies was examined using the Newcastle-Ottawa scale (NOS). Both authors checked the individual articles against the queries of NOS which examines the selection of cohort, comparability of groups, and outcomes. Final scores were given after independent assessments by the authors which ranged from 0 to 9. For follow-up, we considered 1 year of follow-up as adequate for award of points. For comparability, baseline demographics were considered for one point while any other confounder adjusted by the studies was given one point. Disagreements were resolved in consultations with the third author. This was done by comparing the results of each reviewer side-by-side. Discrepancies were identified with reasoning and then structured discussions were conducted by involving a third reviewer. Studies with low NOS scores were not to be excluded from the review.

Two authors sourced information from the studies independently. It was later cross-checked for any errors. Data obtained for this review included: author name, publication year, study type, included patients, type of illness, number of participants, age and gender, disease severity score, percentage of patients with sepsis, timing of RDW measurement, cut-off of RDW, outcomes and follow-up.

### Statistical analysis

We conducted a detailed systematic review of the study outcomes of all included studies. We also extracted all outcome data examining the association between RDW and mortality. Since, data on the length of hospital/PICU stay were not reported by all studies, a meta-analysis was not conducted. For the quantitative analysis, we used “Review Manager” (RevMan, version 5.3). The effect size was combined in the software to generate a pooled odds ratio (OR) with 95% confidence intervals (CI). A *post-hoc* subgroup analysis was done for studies using RDW as a continuous variable and those using specific cut-offs. The choice of meta-analysis model was random-effects. We also quantified the inter-study heterogeneity using the I^2^ index of the software. Values over 50% indicated substantial heterogeneity. Funnel plots were plotted for the meta-analysis. Meta-regression analysis was also conducted with age as the moderator. *P* values <0.05 were considered statistically significant.

## Results

### Search details

**Figure 1:**
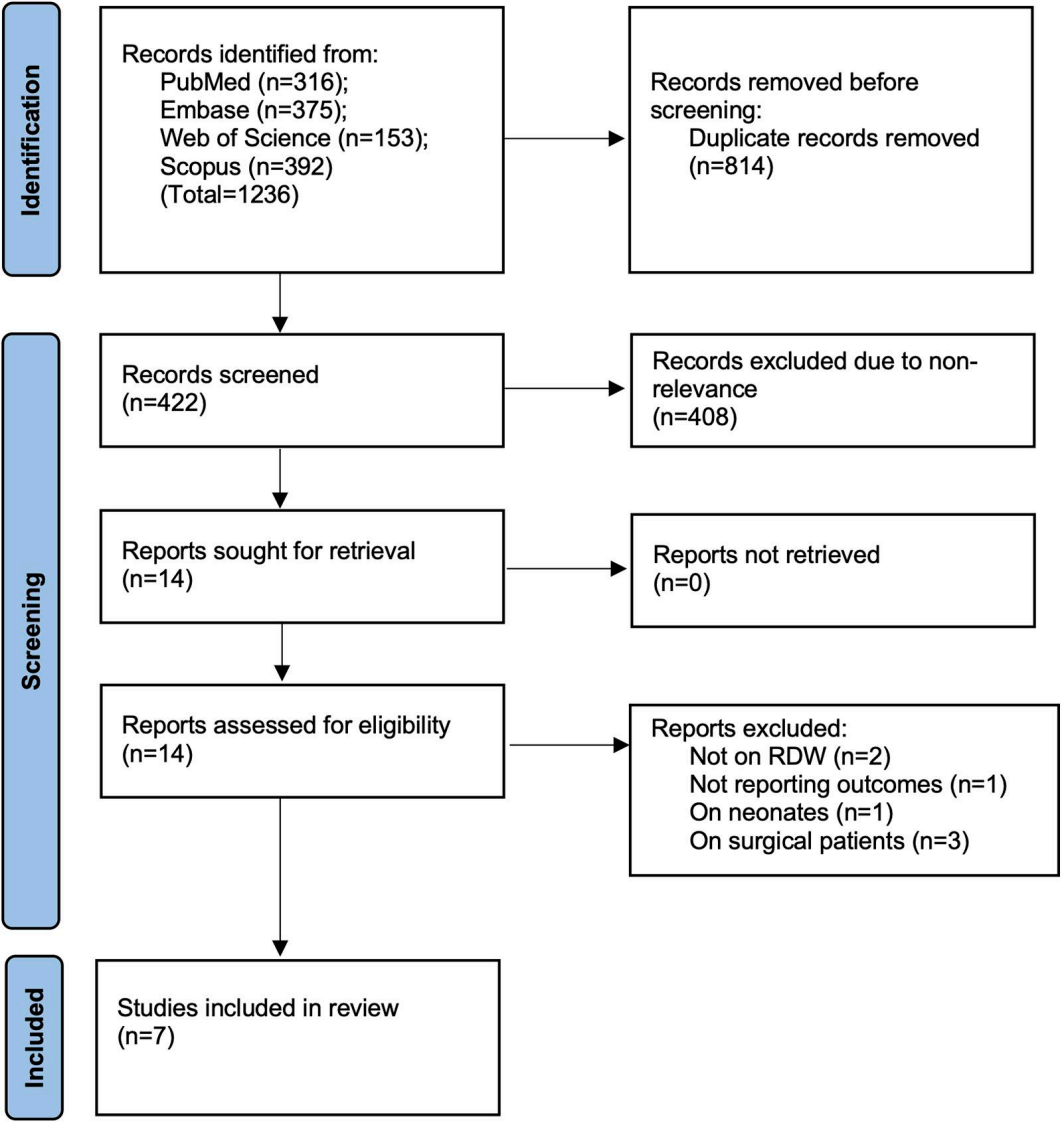
Study selection process.

The number of studies identified in each database and the study selection process are presented in Figure 1. The 1,236 studies identified from the databases initially underwent electronic deduplication. Herein, 814 studies were removed and 422 articles were further screened by the authors. Of these, only 14 were deemed relevant to the review. After completing the full-text analysis, seven studies were included (21–27).

### Details of studies

All information obtained from studies is shown in Table 1. The seven studies were published between 2015 and 2024. The country of origin of all studies was either the USA, China, India, or Korea. Most of them included all PICU patients during the study period. Three studies included either all non-cardiac patients or only influenza or sepsis patients. The combined sample size of all studies was 6,327 pediatric patients. The mean age ranged between 1 and 8 years in the studies. Male patients were more than females in all included studies. The disease severity score varied among the studies. The scores used were either the Pediatric Critical Illness Score, PRISM, or PIM-2. All studies measured RDW on the first day of admission. Three studies used RDW cut-offs which were either 14.5, 14.8 or 15.7 while the remaining used RDW as a continuous variable. All studies reported PICU mortality while only one reported length of hospital stay. Except for two studies which received a NOS score of 7, all of the remaining studies got a score of 8 (Table 2). The inter-reviewer reliability was high with kappa value of 0.85.

**Table 1:** Details of included studies.

Author	Location	Type	Inclusion criteria	Sample size	Mean age (years)	Male (%)	Type of illness	Severity score	Sepsis (%)	RDW timing	Mean RDW (%)	RDW cut-off	Outcomes	Follow-up
Ramby 2015 (21)	USA	R	Consecutive patients admitted to PICU	596	4.4 (1.5–12.9)	53	All types	PIM2: 1.2 (0.8–4.1)	17.4	24 h of PICU admission	14.4 (13.3–15.7)	NR	Mortality, PICU LOS	PICU
Said 2017 (22)	USA	R	All patients admitted to PICU	3,913	7.45 ± 6.67	56.4	Except anemia, malignancy, epilepsy or seizures, organ or tissue transplant recipients	PIM2: −4.59 ± 1.4	NR	On admission	14.1 ± 1.89	14.8	Mortality	PICU
Sachdeva 2018 (23)	India	R	All patients admitted to PICU	101	NR	68	Except hematological disorders	PRISM 12: 22.5 ± 5.7	NR	On admission	NR	15.7	Mortality	PICU
Li 2019 (25)	China	P	Non-cardiac patients admitted to PICU	404	1 (0.3–5)	61.1	Except malignancy, epilepsy or seizures	PCIS: 79.9 ± 13.4	20	24 h of PICU admission	15.2 ± 2.7	NR	Mortality	PICU
Li 2019 (24)	China	R	Sepsis patients admitted to PICU	186	2.2 ± 0.9	58.1	Except blood disorders	PCIS: 77.5 ± 9	100	24 h of PICU admission	15.1 ± 2.7	NR	Mortality	PICU
Kim 2020 (26)	Korea	R	Consecutive patients admitted to PICU	960	1.3 (0.4–4.5)	51.5	All types	PRISM III: 9.04 ± 7.6	NR	24 h of PICU admission	15.6 ± 3.3	14.5	Mortality	PICU
Sun 2024 (27)	China	R	Influenza patients admitted in PICU	167	NR	68.8	Only influenza	NR	19	On admission	NR	NR	Mortality	PICU

RDW, red blood cell distribution width; PCIS, Pediatric critical illness score; PRISM, pediatric risk of mortality score; PIM, Pediatric Index of Mortality; NR, not reported; R, retrospective; P, prospective; PICU, pediatric intensive care unit.

**Table 2:** Risk of bias analysis in the included studies.

Author	Selection of participants	Comparability of groups	Outcome assessment	NOS score
Ramby 2015 (21)	4	2	2	8
Said 2017 (22)	4	1	2	7
Sachdeva 2018 (23)	4	1	2	7
Li 2019 (25)	4	2	2	8
Li 2019 (24)	4	2	2	8
Kim 2020 (26)	4	2	2	8
Sun 2024 (27)	4	2	2	8

NOS, Newcastle Ottawa scale.

### Qualitative analysis

The first study examining the association between RDW and adverse outcomes in PICU was that of Ramby et al. (21) which included all consecutive pediatric patients with varying illnesses. They divided the cohort into RDW quartiles, namely, <13.4%, 13.4–14.3%, 14.4–15.7%, and >15.7% and found that PICU mortality in each group was 3.2%, 4.9%, 5.3%, and 12.9% respectively. The incremental increase in mortality rates seen with higher RDW was statistically significant (*p* < 0.01). Likewise, a statistically significant increase in the length of PICU stay was noted with increasing RDW. Further, on controlling for age, hemoglobin, and PIM-2, the authors noted a statistically significantly higher risk of mortality with per-unit increase in RDW (OR: 1.20 95% CI: 1.07, 1.35). They further conducted a subgroup analysis based on the presence of sepsis and noted that RDW was a predictor of mortality only in patients with sepsis and not without sepsis. The article of Said et al. (22) included all PICU patients except anemia, malignancy, epilepsy or seizures, organ or tissue transplant recipients. The authors found that patients with RDW >14.8% had a statistically significantly higher risk of mortality as compared to those with lower RDW (4.25% vs. 1.95% respectively) (*p* = 0.004). Sachdeva et al. (23) in a cohort study in an Indian hospital examined all critically ill patients except for those with hematological disorders admitted to the PICU. They found that high RDW (>18.04%) was associated with a significant increase in the risk of mortality (*p* < 0.04) but the risk was not significant in those with RDW between 15.7–18.04% (*p* = 1). They also reported that the optimal RDW cut-off for predicting mortality was 18.6% which had a sensitivity and specificity of 90.9% and 70.8% respectively. Li et al. (24) examined only PICU patients with sepsis and found that RDW was not predictive of mortality (OR: 0.95 95% CI: 0.65, 1.40). Another study of Li et al. (25) which included only non-cardiac patients admitted to the PICU found that high RDW had a tendency of higher mortality but results were not statistically significant (OR: 1.79 95% CI: 0.98, 3.26). They also showed that the optimal RDW cut-off to predict mortality was ≥15.52% which had a sensitivity of 75.76% and specificity of 63.61%. Kim et al. (26) examined all PICU patients with varying illnesses and found that after adjustment of age, sex, and C-reactive protein, RDW was predictive of mortality (OR: 2.32 95% CI: 1.35, 3.98). Lastly, Sun et al. (27) examined only influenza patients admitted to the ICU. They found that after multivariate adjustment of data RDW was predictive of mortality (OR: 1.38 95% CI: 1.11, 1.71).

### Quantitative analysis

**Figure 2:**
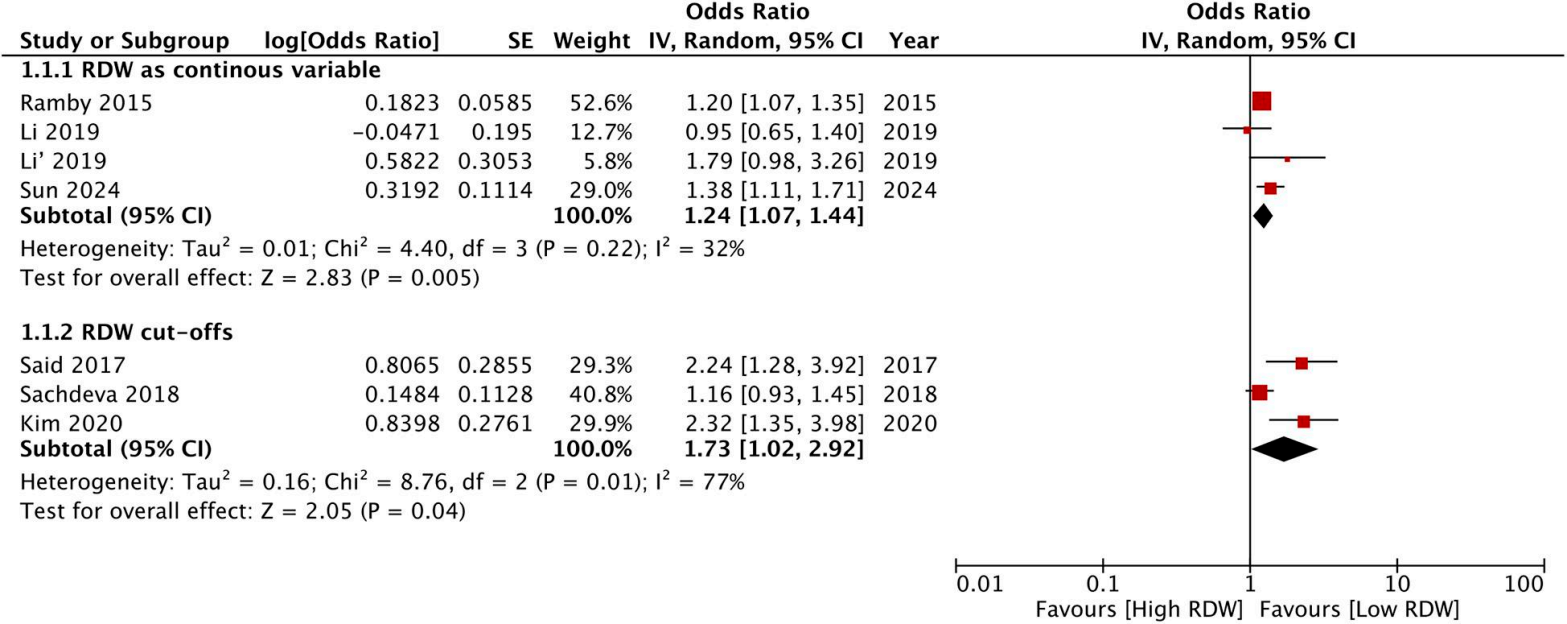
Meta-analysis of the association between RDW and PICU mortality of critically ill pediatric patients. Subgroup analysis was done for RDW as continuous or categorical variable.

**Figure 3:**
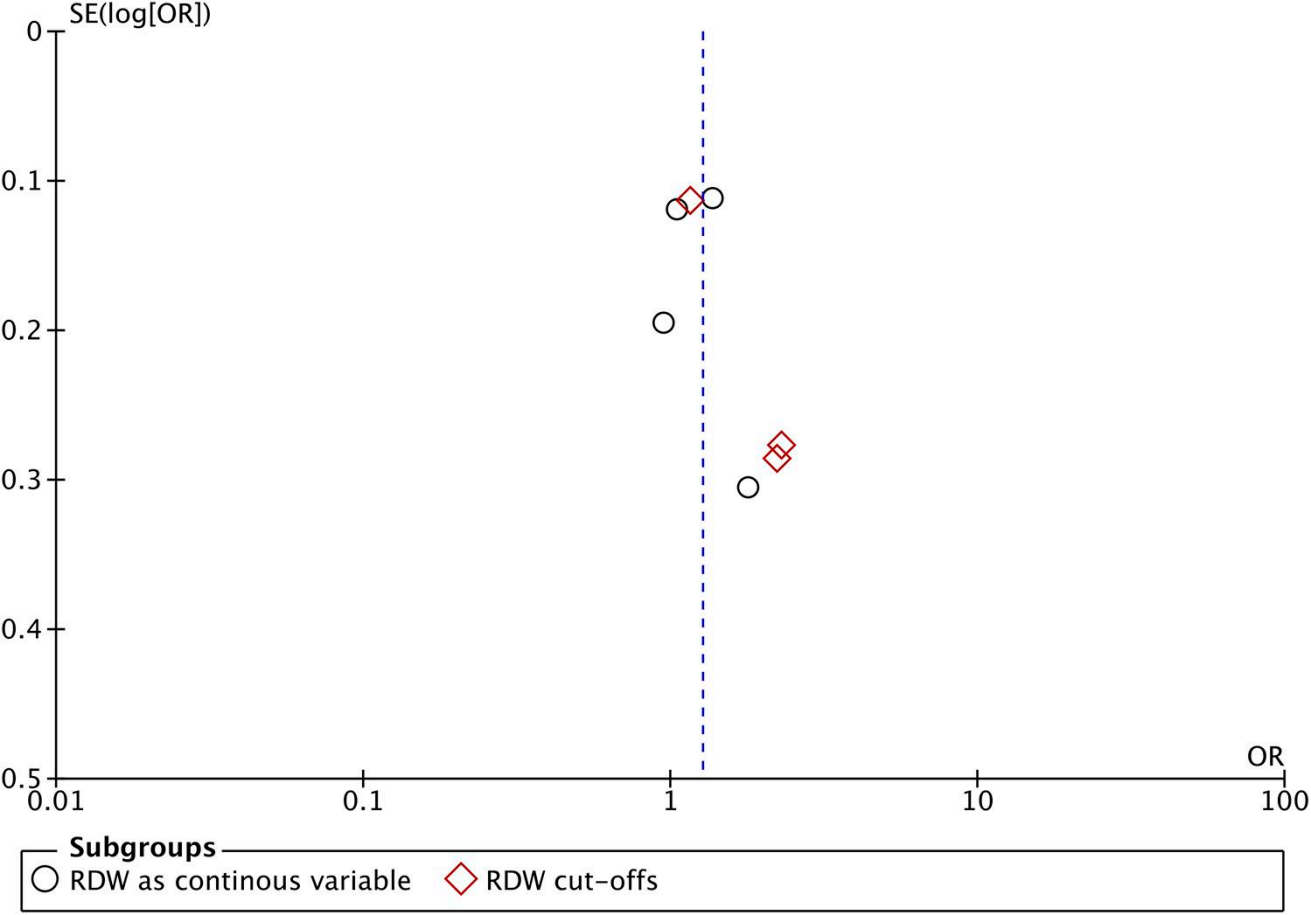
Funnel plot for the meta-analysis.

**Figure 4:**
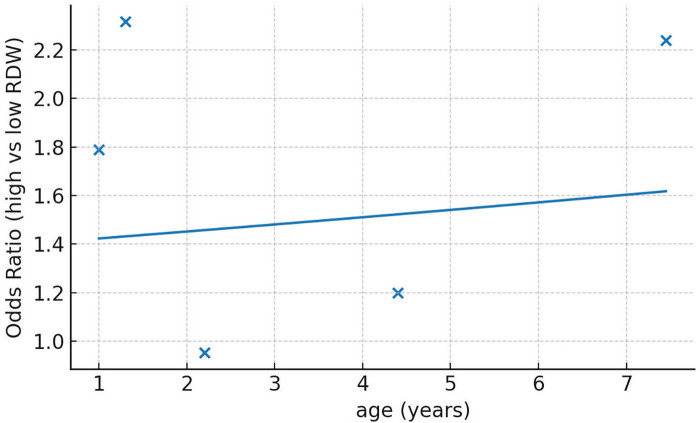
Meta-regression plot for assessing the effect of age on mortality with high vs. low RDW.

We segregated studies based on how RDW was used to assess the risk of PICU mortality. Four studies used RDW as a continuous variable while three studies reported specific RDW cut-offs and divided the sample into high and low RDW groups. On pooled analysis of studies which used RDW as a continuous variable, we noted that an incremental increase in RDW was associated with a statistically significant increased risk of mortality (OR: 1.24 95% CI: 1.07, 1.44). Inter-study heterogeneity was low (I^2^ = 32%) (Figure 2). Pooled analysis of three studies using RDW as a categorical variable showed that higher values of RDW were associated with significantly higher risk of mortality (OR: 1.73 95% CI: 1.02, 2.92). Inter-study heterogeneity was high in this case (I^2^ = 77%) (Figure 2). No obvious asymmetry was noted on the funnel plot (Figure 3). Meta-regression analysis showed no significant relationship between mean age and the mortality risk effect of high vs. low RDW (Beta: 0.0198 95% CI: −0.124, 0.163 *p* = 0.79) (Figure 4).

## Discussion

Mortality is the worse outcome noted in a PICU and its incidence varies significantly based on healthcare setups, disease severity and treatment protocols (1, 4). Pediatric patients in critical condition often present with significant deviations from the body's normal balance and variations of relevant markers can be used to predict prognosis (4). One of the earliest markers used in PICU was the PRISM score which originally included about 14 variables including blood pressure, heart rate, respiratory rate, arterial blood gas values, Glasgow coma scale, and several other serum measures (28). The revised version, PRISM III is also a highly accurate mortality prediction model which is derived from 17 physiological variables which are further subdivided in 26 ranges (29). Another marker commonly used in the PICU is PIM-2 which is a slightly simpler model based on ten different variables which includes blood pressure, pupillary reaction, blood gas values, reason for admission, and diagnosis. It has also shown high discriminative ability between survivors and death at the PICU (30). Likewise, there are numerous other scoring models like PELOD, pediatric sequential organ failure assessment and updates of prior models like PIM-3 and PRISM-IV which aim to accurately predict mortality (31, 32). A major limitation of these models is the need for lengthy calculations and several measurements which can not only be time-consuming but also difficult in high-pressure and resource-limited settings. While we agree that these models are currently indispensable in PICUs and cannot be replaced, there is still a need for simpler, easy to use biomarker that can provide a preliminary assessment of mortality risk in critically ill pediatric patients. There have been several such simpler markers explored in literature like body mass index, N-terminal brain natriuretic peptide precursor, lactate clearance, C-reactive protein, serum calcium, and RDW but with varying results (25, 33–36). To the best of our knowledge, one of the most commonly investigated markers is RDW.

RDW is routinely used in the differential diagnosis of anemia but a large body of evidence now shows its significance in predicting prognosis in a variety of clinical disorders (10–16). Furthermore, it is a predictor of all-cause and cause-specific mortality (cancer-related, respiratory-related, cardiovascular-related) in the general population as well (37). The wide-spanning prognostic ability of RDW has been attributed to its relationship with anemia, inflammation, and oxidative stress all of which could lead to higher mortality risk (10). Anemia is commonly seen in critically ill patients and could be related to phlebotomy, coagulopathy, pathogen-induced hemolysis, and nutritional deficiency. This leads to ineffective erythropoiesis and release of immature RBC explaining the variability in RDW. Anemia itself reduces oxygen delivery to tissues, worsening outcomes in critically ill patients. Furthermore, anemic patients have significantly poorer outcomes as compared to those with normal hemoglobin values (38). Secondly, it is known that inflammatory cytokines affect the maturation of red blood cells (RBC) by inhibiting production and response to erythropoietin, leading to diminished iron metabolism and reduced RBC survival causing increased RDW (39, 40). A strong correlation between inflammatory markers like interleukin-6, C-reactive protein, and erythrocyte sedimentation rate has been noted with RDW (41). Additionally, tumor necrosis factor leads to hypoferremia which causes erythrophagocytosis. Cytokines also lead to deformities in RBC membranes suppressing erythrocyte maturation. This leads to the production of larger reticulocytes causing an increase in RDW. The antioxidant defense system in critically ill children is not fully mature, making them more susceptible to oxidative stress. When compared to adults, neonatal RBCs have reduced quantities of important antioxidants such as glutathione, catalase, and superoxide dismutase. Glutathione shortage restricts the neutralization of reactive oxygen species, whereas low catalase activity affects the breakdown of hydrogen peroxide into water and oxygen. This immaturity causes higher oxidative damage to erythrocyte membranes, hemoglobin oxidation, and a shorter RBC lifespan. The bone marrow adjusts by producing more immature, variable-sized red cells, increasing RDW. During critical illness, oxidative stress is exacerbated by hypoxia, infection, and systemic inflammation, overloading the already low antioxidant capacity. This leads to cellular damage, poor oxygen supply, multiorgan dysfunction, and, ultimately, greater mortality in critically ill pediatric patients. Thus, RDW can be considered to be reflective of inflammatory process which is common to several diseases leading to the prediction of worse outcomes (10, 42, 43).

Our study presents a detailed systematic review and meta-analysis exploring the ability of RDW to predict mortality in PICU. On qualitative assessment of studies, it was noted that despite inclusion of pediatric patients with varying illnesses, most studies noted a positive association between high RDW and death in the PICU. There were just two studies, one on only sepsis patients (24) and the other on only non-cardiac patients (25) which noted no association between RDW and mortality. Of these studies, the study of Li et al. (25) still noted a tendency of higher mortality with RDW but the results did not achieve statistical significance. One possible reason for the non-significant result could be the small sample size of these studies. On pooled analysis of data, we noted that higher RDW was significantly associated with a higher risk of mortality. Importantly, the association was persistent for studies using RDW as a continuous variable as well as a categorical variable. This indicates that even per per-unit increase in RDW can be a predictor of mortality in the PICU but the association may be stronger when a specific cut-off is determined. But the optimal cut-off for predicting mortality with RDW is still unclear. On one hand, Li et al. (25) found that a value of 15.52% had a sensitivity of 75.76% and specificity of 63.61% while Sachdeva et al. (23) showed that a value of 18.6% had a sensitivity and specificity of 90.9% and 70.8% respectively. Given the small number of studies, there is a need for further research assessing the optimal cut-off for RDW in determining mortality in the PICU.

A prior review has also reported that RDW can be a predictor of mortality in pediatric patients (44). However, our review is significantly different as we included only adjusted data which increases the reliability of the results. Secondly, we segregated the data of RDW as continuous and categorical variables and performed a subgroup analysis for the same which was not conducted in the earlier study. Thirdly, a detailed qualitative analysis was also conducted which is missing in the previous study. Our results are also similar to those reported in adult populations. In a systematic review of 32 studies, Luo et al. (45) have shown that RDW when used as a continuous and categorical variable was predictive of mortality in critically ill adult patients. Another recent study by Peng et al. (46) examined 26,818 mixed critically ill patients from the MIMIC-III database and showed that RDW values were positively associated with 30-day, 90-day, 365-day, and 4-year all-cause mortality in such patients. Danki et al. (47) have examined data from the eICU Collaborative Research Database, which included 16,423 septic patients. They found that patients with RDW ≥15% had a two-fold higher risk of mortality as compared to those with RDW <15%. However, per-unit increase in RDW was associated with only a 16% increase in ICU mortality and an 18% increase in hospital mortality. They noted that RDW had diagnostic performance equivalent to the Sequential Organ Failure Assessment (SOFA) score and Acute Physiology and Chronic Health Evaluation (APACHE) IV score, which are commonly used in adult critically ill patients. Indeed, such comparisons between RDW and commonly used pediatric scores like PRISM, PIM and PELOD have not been reported in the literature. Till such evidence is made available, the diagnostic performance of RDW vis-à-vis other scores cannot be gauged. Therefore, there is a need for further research not only examining the predictive value of RDW but also comparing it with standard markers so that high-quality evidence is available for pediatricians.

Several limitations can be noted for our review. Firstly, the availability of studies was not high. We could include only seven studies despite a detailed literature search. The paucity of studies precluded a detailed subgroup or meta-regression analysis which could have provided further details on the predictive ability of RDW for specific patient populations. We also acknowledge the high heterogeneity in the meta-analysis which is primarily due to the varied patient populations in the included studies. The cohorts spanned mixed illnesses like non-cardiac, influenza, sepsis, etc. and the studies failed to report segregated data based on the specific diseases. Therefore, it was not possible to sort patients with similar disease or disease severity given the lack of raw data from the studies. Furthermore, the disease severity scores reported by the studies also varied which prevented segregation for a subgroup analysis. Hence, a detailed systematic review was also performed along with the meta-analysis. Another limitation of the review is that we could only assess PICU mortality rates as data on long-term mortality and other outcomes like PICU stay were not reported evenly by the studies. PICU or length of hospital stay is an important outcome that has significant clinical implications especially in low-resource settings. Given the limited data, the generalizability of RDW is therefore restricted at this point. Another factor of significance is the change in RDW during the treatment. All studies measured RDW on the first day of admission and it is not known how does change in RDW affects prognosis. We also excluded studies on neonates as they differ significantly from older children in their physiology due to ongoing developmental changes. Neonates have immature organ systems, higher metabolic rates, and different fluid and electrolyte balances and hence a separate investigation is needed to assess the prognostic ability of RDW in such populations. Furthermore, mortality in a PICU depends on a wide array of covariates. Some studies adjusted for a larger number of confounders while others had minimal or unclear adjustments. Despite most studies reporting adjusted data, all possible confounders were not adjusted and it is possible that outcomes may have been skewed. Lastly, the search strategy used in the review by excluding keywords like “PICU”, “pediatric intensive care” and “critical care” may not be focused to the research question. Use of these keywords in the search may have produced a more targeted search strategy allowing easy replicability.

## Conclusions

This systematic review and meta-analysis of literature shows that RDW may have a role in predicting mortality in the PICU. However, the high heterogeneity in the meta-analysis especially due to the varied population included, limits the generalization and clinical applicability of the results. Further research on the specific pediatric PICU population is needed for more robust results. Also, future studies should decipher the optimal cut-off of RDW and compare its predictive ability with standard scoring systems for routine clinical application.

## Data Availability

Publicly available datasets were analyzed in this study. This data can be found here: PubMed, Embase, Scopus, and Web of Science.
